# Skeletal and Bone Mineral Density Features, Genetic Profile in Congenital Disorders of Glycosylation: Review

**DOI:** 10.3390/diagnostics11081438

**Published:** 2021-08-09

**Authors:** Patryk Lipiński, Karolina M. Stępień, Elżbieta Ciara, Anna Tylki-Szymańska, Aleksandra Jezela-Stanek

**Affiliations:** 1 Department of Pediatrics, Nutrition and Metabolic Diseases, The Children’s Memorial Health Institute, 04-730 Warsaw, Poland; A.Tylki@ipczd.pl; 2 Adult Inherited Metabolic Diseases, Salford Royal NHS Foundation Trust, Salford M6 8HD, UK; kstepien@doctors.org.uk; 3 Department of Medical Genetics, The Children’s Memorial Health Institute, 04-730 Warsaw, Poland; e.ciara@ipczd.pl; 4 Department of Genetics and Clinical Immunology, National Institute of Tuberculosis and Lung Diseases, 01-138 Warsaw, Poland; jezela@gmail.com

**Keywords:** congenital disorder of glycosylation, growth, skeletal dysplasia, cartilage, joint, bone markers, osteoporosis

## Abstract

Congenital disorders of glycosylation (CDGs) are a heterogeneous group of disorders with impaired glycosylation of proteins and lipids. These conditions have multisystemic clinical manifestations, resulting in gradually progressive complications including skeletal involvement and reduced bone mineral density. Contrary to PMM2-CDG, all remaining CDG, including ALG12-CDG, ALG3-CDG, ALG9-CDG, ALG6-CDG, PGM3-CDG, CSGALNACT1-CDG, SLC35D1-CDG and TMEM-165, are characterized by well-defined skeletal dysplasia. In some of them, prenatal-onset severe skeletal dysplasia is observed associated with early death. Osteoporosis or osteopenia are frequently observed in all CDG types and are more pronounced in adults. Hormonal dysfunction, limited mobility and inadequate diet are common risk factors for reduced bone mineral density. Skeletal involvement in CDGs is underestimated and, thus, should always be carefully investigated and managed to prevent fractures and chronic pain. With the advent of new therapeutic developments for CDGs, the severity of skeletal complications may be reduced. This review focuses on possible mechanisms of skeletal manifestations, risk factors for osteoporosis, and bone markers in reported paediatric and adult CDG patients.

## 1. Introduction

**Figure 1:**
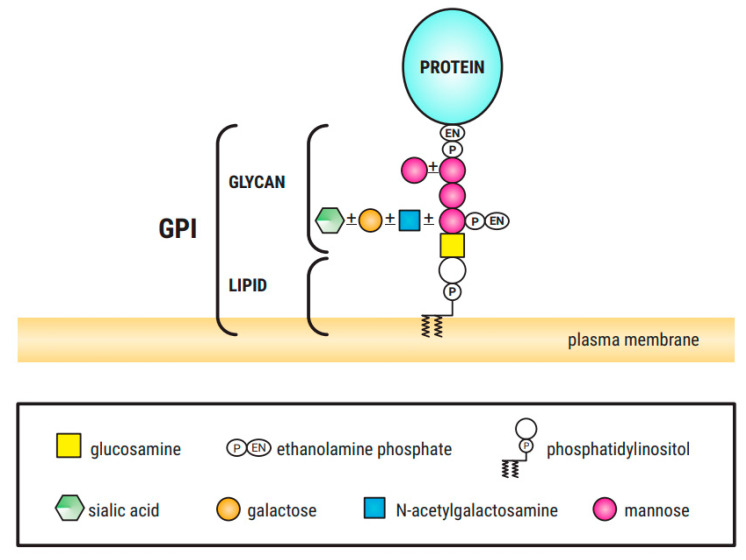
GPI structure, consisting of the conserved core glycan, phosphatidylinositol and glycan side chains ([3], own modification).

Congenital Disorders of Glycosylation (CDGs) are a clinically heterogenous group of over 150 diseases caused by defects in different steps of glycan metabolism pathways [1,2]. These genetic disorders are characterized by an impaired synthesis and attachment of glycans to glycoproteins and glycolipids, and impaired synthesis of glycosylphosphatidylinositol (GPI) (Figure 1) [1]. These disorders are categorized into (a) protein *N*-glycosylation defects; (b) protein *O*-glycosylation defects; (c) glycolipid and GPI anchor synthesis defects and (d) multiple glycosylation pathways and other pathways defects [2].

Among the different glycosylation defects, impaired protein *N*-glycosylation is the most common. Glycan processing (remodeling) takes place in the endoplasmic reticulum (ER) and Golgi apparatus [2]. *O*-glycosylation occurs in the Golgi apparatus and no lipid-linked intermediates are involved. In contrast to *N*-glycosylation, the process involves only the sequential addition of monosaccharides without remodeling [2,4]. Abnormalities in the synthesis of *O*-linked *N*-acetylglucosamine, galactose (Gal), mannose (Man), glucose (Glc) and fucose (Fuc) glycans have also been described [5]. GPI is a phosphoglyceride (Figure 1) that acts as a membrane anchor of proteins to the outer leaflet of the cellular membrane. GPI-anchor biosynthesis defects (GPI-BD) are categorized by an inappropriate GPI-anchors biosynthesis or modification (protein attachment, structural remodeling at both glycan and parts of GPI). Most of the CDGs have autosomal recessive pattern of inheritance, but autosomal dominant and X-linked forms have also been described. To date, about 80 different entities have been described in correlation with congenital disorder of glycosylation or CDG-like diseases. Although some genotype–phenotype correlation has been proposed, there is a significant phenotypic variability within the same genotype.

Glycosylation is key for a variety of posttranslational biological processes including main components of the hormone cascades regulating growth and metabolism [5,6]. As a result, CDG patients present with very diverse clinical phenotypes, with multi-system involvement [4,5,6,7]. These include cognitive impairment, neurological (epilepsy, hypotonia, ataxia and polyneuropathy), ophthalmological, skeletal, cardiac, hepatic, haematological and endocrinological phenotypes [1,2,7,8].

The mechanisms of bone disease in CDGs seem to be multifactorial. Malabsorption and liver dysfunction lead to a poor nutritional state which impacts the growth hormone-insulin–like growth factor (GH/IGF) axis and results in a short stature [9,10]. Given that patients with CDGs manifest their skeletal abnormalities in childhood, one may speculate that they are attributed to the impaired glycosylation in the bone tissue in the early stages of life.

So far, there have been no therapies targeting specific organ such as eye, kidney, heart, muscles or skeleton, with only supportive management being available [10].

This review describes skeletal manifestations and bone mineral density abnormalities in paediatric and adult CDG patients with the aim to discuss the heterogeneity of clinical manifestations and correlate the genetic data (causative genes) with observed skeletal phenotype to evaluate the potential mechanism of mutational effect on the phenotype.

## 2. Skeletal Abnormalities

### 2.1. Skeletal Characteristics of PMM2-CDG

**Figure 2:**
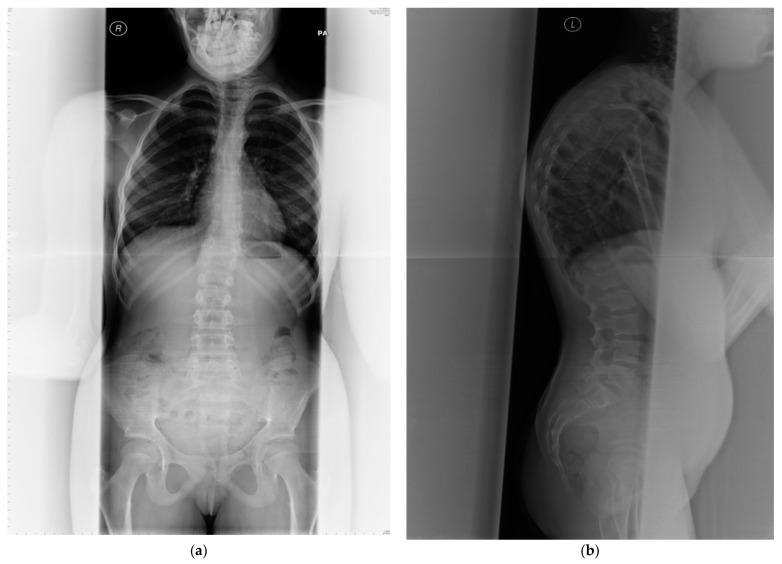

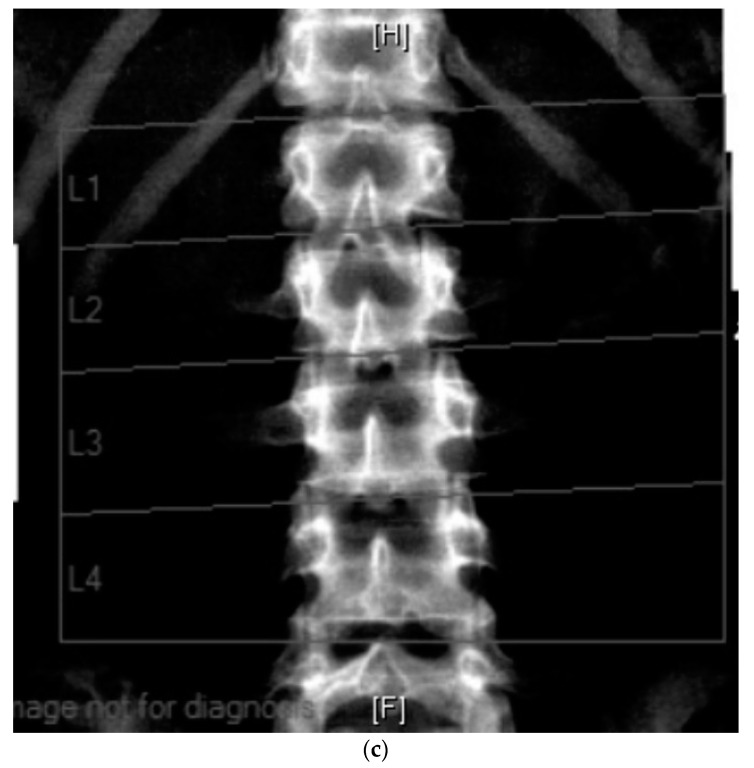
(**a**) Anterior posterior view—whole spine x-ray in a 25-year-old female patient affected with PMM2-CDG; bone mineral density is below expected for her age (T score −2.6, z-score −2.6); (**b**) lateral view; (**c**) radiological features of her lumbar spine: there is a double mild curve scoliosis and no rotational deformity. All pedicles are visualized. There is impression of slight upper hyperkyphosis and impression of multilevel Schmorl’s nodes.

PMM2-CDG is the most prevalent disorder of abnormal glycosylation of *N*-linked oligosaccharides leading to endocrine abnormalities including dysfunction of IGFBP3 and an acid-labile subunit in the IGF pathway which result in short stature [11]. Growth failure is commonly observed in children with PMM2-CDG [7,12,13,14,15,16,17]. Despite normal serum calcium, phosphate and magnesium concentrations, osteopenia/osteoporosis are frequently demonstrated in this condition since childhood (Table 1) [8,9,10,11,12,13,14]. Skeletal abnormalities are common, although under-diagnosed [17], often leading to kypho/scoliosis, severe spinal cord deformity and vertebral compression fractures [7] (see Figure 2). Regular orthopaedic assessment and intervention are required if scoliosis becomes evident, with cervical spine x-rays in neutral, flexion, and extension to assess for atlantoaxial instability. Fractures are common and appear to heal normally. Skeletal dysplasia is not a typical feature of PMM2-CDG but has been reported before (Table 1 and Table 2). Joint contractures are relatively common and affect patients’ quality of life.

### 2.2. Skeletal Dysplasia in Other CDGs

Contrary to PMM2-CDG, skeletal dysplasia was well characterized in other CDGs, including ALG12-CDG, ALG3-CDG, ALG9-CDG, ALG6-CDG, PGM3-CDG, CSGALNACT1-CDG, SLC35D1-CDG and TMEM165-CDG (Table 1).

The combination of features observed in patients with ALG12-CDG, including interphalangeal dislocations, talipes equinovarus, rhizomelic limb shortening, midface hypoplasia, short metacarpals, and a horizontal acetabular roof, resembled the features of pseudodiastrophic dysplasia (Table 1) [21,22]. Pseudodiastrophic dysplasia (264180) is characterized by rhizomelic shortening of the limbs and severe clubfoot deformity, in association with elbow and proximal interphalangeal joint dislocations, platyspondyly, and scoliosis [23,24,25].

The features of patients with ALG9-CDG reported by Tham et al. were similar to those reported by Gillessen-Kaesbach and Nishimura and to some extent overlapping with those of individuals with ALG3-CDG and ALG12-CDG (Table 1 and 2) [27,28,29,30].

Most patients affected with PGM3-CDG showed skeletal abnormalities (Table 1 and Table 2). Scoliosis has been reported as the mildest manifestation of the PGM3-related skeletal phenotype [33]. Two patients described by Stray-Pedersen et al. (2014) presented with a recognizable skeletal dysplasia resembling Desbuquois dysplasia (#251450) [34]. This condition is an autosomal recessive osteochondrodysplasia characterized by severe prenatal and postnatal growth retardation (stature less than −5 SD), short extremities (rhizomelic and mesomelic shortening), shortened tubular bones with metaphyseal flaring, an exaggerated trochanter minor of the proximal femur (monkey-wrench malformation), advanced bone age, joint laxity, and progressive kyphoscoliosis (Table 2) [35,36].

Skeletal dysplasia was also reported as the main clinical feature of patients with COG7-CDG, COG1-CDG and COG8-CDG (Table 1). While patients with COG8-CDG displayed severe skeletal dysplasia, COG1-CDG and COG8-CDG patients had a significantly milder clinical presentation (Table 2) [37,38,39,40].

### 2.3. Joint and Cartilage Involvement in CDG

In two patients with typical Schneckenbecken dysplasia (# 269250), Rautengarten et al. found biallelic mutations in both alleles of *SLC35D1* (Table 1 and Table 2) [43]. Schneckenbecken dysplasia (#269250) is a perinatally lethal skeletal dysplasia characterized by the distinctive, snail-like appearance of the ilia, thoracic hypoplasia, severe flattening of the vertebral bodies and short, thick long bones [44].

Among genes implicated in Ehlers-Danlos syndrome (EDS), biallelic mutations in *B4GALT7* (encoding galactosyltransferase-I) have been described in patients affected with the progeroid form of EDS presenting with radioulnar synostosis, diffuse osteopenia, splaying of the ribs, broad thumbs and long fingers, long and overriding toes, equinovarus deformities, single palmar crease, and hypermobile joints (Table 1 and Table 2) [45,46,47,48].

Short stature, generalized osteopenia/osteoporosis and epi- and metaphyseal dysplasia were reported in patients with TMEM165-CDG (Table 1) [6,50].

### 2.4. GPI-Biosynthesis Defects (GPI-BD)

Defects in the glycosylphosphatidylinositol (GPI) biosynthesis pathway have unique skeletal manifestations. GPI-anchor biosynthesis can be divided into the synthesis stage (responsible genes: *DPM1, DPM2, DPM3, MPDU1, PIGA, PIGB, PIGC, PIGF, PIGG, PIGH, PIGL, PIGM, PIGN, PIGO, PIGP, PIGQ, PIGV, PIGW, PIGX, PIGY*) and the transamidase + remodelling stage (responsible genes: *GPA1, MPPE1, PGAP1, PGAP2, PGAP3, PIGK, PIGS, PIGT, PIGU*) [51]. Abnormal digit morphology, absent distal phalanges, aplasia/hypoplasia of fingers, short digit, broad finger and broad toe, clubbing, and clinodactyly were more frequently observed in the synthesis group (Table 1) [52,53,54,55,56,57]. On the other hand, osteopenia was more frequently observed in the transamidase + remodelling group [53].

Brachytelephalangy was consistently found in all the reported PIGV-CDG patients (Table 2) [52]. It was also found in most of the reported patients with PIGO-CDG (Hyperphosphatasia with mental retardation syndrome 2, HPMRS2, #614749) [58,59]. This skeletal finding could lead to the suspicion of Coffin-Siris syndrome given the neurological findings in both syndromes, i.e., delayed psychomotor development, hypotonia and expressive language delay. Coffin-Siris syndrome in its typical form (CSS1, #135900) characterized by aplasia or hypoplasia of the distal phalanx and hypoplastic, aplastic nails (even absent fifth fingernail in some patients), developmental or cognitive delay of varying degree, distinctive facial features, hypotonia, hirsutism/hypertrichosis, and congenital malformations of the cardiac, gastrointestinal, genitourinary, and/or central nervous systems [60].

### 2.5. Genotype–Phenotype Correlation

The genetic background of CDG is highly variable and includes various genes. In Table 2, we report the clinical and molecular findings of CDG patients with skeletal abnormalities. Most of patients were compound heterozygotes for autosomal recessive hypomorphic mutations and homozygotes are rather sporadic or family related cases. It is difficult to draw any strong conclusions regarding the possible genotype–phenotype correlation in such a heterogenic group.

For patients with homozygous mutations, we were able to give some conclusions regarding, i.e., *CSGALNACT1* and *SLC35D1* genes. Knockout of the orthologous mouse *CSGALNACT1* indicates that the protein is necessary for normal cartilage development and aggrecan metabolism [61]. Mutations in *CSGALNACT1* are associated with a mild skeletal dysplasia and joint laxity [41]. The clinical significance of the described variant c.791A > G (p.Asn264Ser) is defined as likely pathogenic.

Hiraoka et al. created Slc35d1-deficient mice that present with a lethal form of skeletal dysplasia (severe shortening of limbs and facial structures) with short, sparse chondroitin sulphate chains caused by a defect in chondroitin sulphate biosynthesis [62]. Epiphyseal cartilage in these homozygous mutant mice showed a decreased proliferating zone with round chondrocytes, scarce matrices and reduced proteoglycan aggregates. Variant c.125delA in SLC35D1 was reported only once, and its significance has been defined as pathogenic (https://www.ncbi.nlm.nih.gov/clinvar/RCV000001182, accessed on 27 June 2021).

Skeletal and/or bone mineral density abnormalities are common features of all CDG types. Osteoporosis and/or osteopenia, growth failure, skeletal dysplasia and joint abnormalities were described (Table 1 and Table 2). Genotype–phenotype correlation is impossible due to gene heterogeneity, diversity of individual markers and compound heterozygosity in most described cases (Table 2).

Contrary to PMM2-CDG, skeletal abnormalities are well characterized in other CDG. There is a group of CDGs, including ALG12-CDG, ALG3-CDG, ALG9-CDG, ALG6-CDG, PGM3-CDG, CSGALNACT1-CDG, SLC35D1-CDG, and TMEM-165 with well-defined skeletal dysplasia. In some of them (ALG12-CDG, ALG3-CDG, ALG9-CDG, PGM3-CDG, SLC35D1-CDG), a severe prenatal-onset skeletal dysplasia was observed and associated with an early death. Among them, ALG12-CDG, ALG3-CDG and ALG9-CDG represent distinct entities involved in primary lipid-linked oligosaccharide (LLO) synthesis. These three mannosyltransferases function in direct sequential order, adding mannose moieties to LLO. On the other hand, phosphoglucomutase 3 (PGM3) is required for the biosynthesis of uridine diphosphate *N*-acetylglucosamine (UDP-GlcNAc), a precursor for both *N*-linked and *O*-linked glycosylated proteins. These facts could explain the severe phenotype of reported skeletal abnormalities.

Glycosylation is essential for proteins involved in the development of cartilage and bone and in skeletal patterning pathways. Two of the glycosylation defects, TMEM165-CDG and CSGALNACT1-CDG, are characterized by skeletal dysplasia and abnormal cartilage development. It has been also observed in an experimental study by Bammens et al. (2015), that the TMEM165-deficient zebrafish model exhibited phenotypic patterns of bone dysplasia and abnormal cartilage development similar to the major clinical findings reported in three patients with a homozygous splice mutation [63].

Two other CDGs, CSGALNACT1-CDG and SLC35D1-CDG, are caused by defects in genes responsible for glycosaminoglycans biosynthesis.

*CSGalNAcT1* (chondroitin sulphate *N*-acetylgalactosaminyltransferase-1) gene codes the CSGalNAcT-1 protein that is the main enzyme in the biosynthesis of sulphated glycosaminoglycans chondroitin and dermatan sulphate in cartilage [42]. Biallelic loss-of-function mutations in *CSGALNACT1* gene results in reduced CSGalNAcT-1 activity leading to altered levels of chondroitin, dermatan, and heparan sulphate and cause a mild skeletal dysplasia with joint laxity and advanced bone age, CSGALNACT1-CDG [42].

Another gene, *SLC35D1* gene, encodes the protein that transports substrates needed for glucuronidation and chondroitin sulphate biosynthesis [62]. The protein resides in the endoplasmic reticulum, and transports both UDP-glucuronic acid and UDP-*N*-acetylgalactosamine from the cytoplasm to the endoplasmic reticulum. In humans, loss of function mutation in this gene cause Schneckenbecken dysplasia associated with perinatal lethal skeletal dysplasias [62].

### 2.6. Risk Factors for Osteoporosis in CDGs

Hormonal dysfunction, resulting from hypogonadotrophic hypogonadism, is a risk factor for reduced bone mineral density in females with CDGs [7,64,65]. It requires treatment with oestrogen to avoid osteoporosis; however, it increases the risk of thrombotic events and must be dosed carefully.

Gastrointestinal problems and malabsorption may lead to hypovitaminosis D. It is therefore worth considering screening for common causes of chronically decreased vitamin D such as celiac disease [65].

Bone deformities and chronic pain result in limited mobility and wheelchair use. Low BMI and disuse sarcopenia are frequent clinical features of patients with CDGs and, in the long term, they may cause low bone mineral density and recurrent fractures.

### 2.7. Bone Markers

Historically, this GPI-linked enzyme, has been used to assign glycosylphosphatidylinositol biosynthesis defects (GPI-BD) to the phenotypic group termed hyperphosphatasia with mental retardation syndrome (HPMRS) and to distinguish them from another subset of GPI-BD—multiple congenital anomalies hypotonia seizures syndrome (MCAHS) [51]. However, with the increasing number of individuals diagnosed with a GPI-BD, hyperphosphatasia has been shown to be a variable feature and not more useful for GPI-BD distinguishing.

Bone-specific alkaline phosphatase activity measured in serum is an indicator of osteoblastic activity.

Serum and urinary deoxypyridinoline and pyridinoline, cross-linking amino acids that stabilize collagen chains within the extracellular matrix, can also be measured when screening for reduced bone mineral density in PMM2-CDG disorders [10]. Bone markers of formation (P1NP) or resorption (CTX) measurement in CDGs requires further clinical studies to better understand what factors are likely to affect their concentration in these conditions. All the bone markers should be interpreted in the context of symptoms, age, gender, limited physical activity including wheelchair use, and diet.

Transferrin glycosylation has been the main biomarker for CDG screening and monitoring of the response to treatment. The analysis of protein *N*-glycosylation profile is widely used in CDGs and other conditions [66].

There is a scope for using *N*-glycans in monitoring of bone disease progression and further research is warranted to confirm the preliminary findings [67].

**Figure 3:**
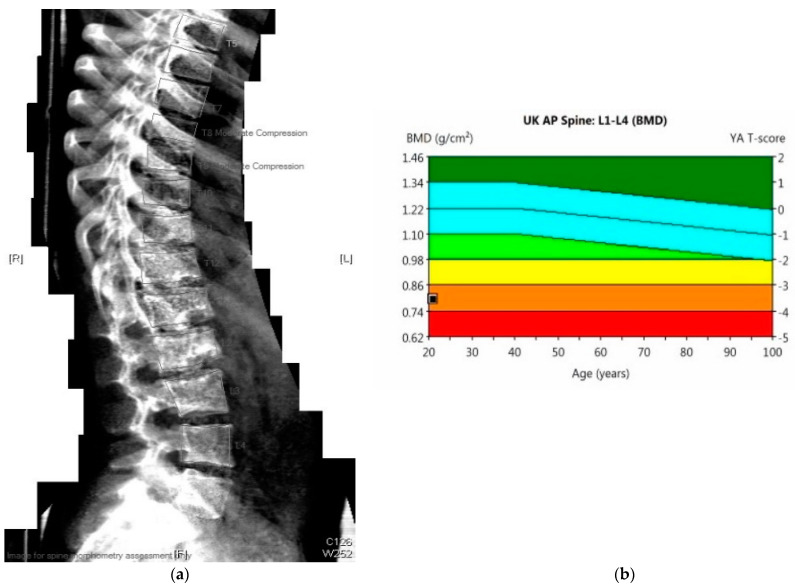
(**a**) 23-year-old male with PMM2-CDG (BMI 17 kg/m^2^). DEXA scan: Results: bone mineral density below expected range for age. Evidence of moderate compression at T8 and T9 vertebrae. (**b**) DEXA scan: Lumbar spine (L1–L4): T-score −3.5, Z-score −3.5, bone mineral density 0.8 g/cm^3^.

The effectiveness of supportive management can be evaluated by DEXA scan (Figure 3), a gold standard method for monitoring the progression of bone mineral density decline. The whole spine x-ray (Figure 2) can be used for identification of bone deformities and fractures. QCT however is the sensitive quantitative method used to assess the bone mineral density. Body composition has become increasingly utilized in Inherited Metabolic Disorders field and provides additional information about the bone and muscle involvement.

Importantly, a multidisciplinary approach with pain specialist, rheumatologist, endocrinologist, metabolic specialist, physiotherapist and dietician is key to successful management of bone health in this group of disorders.

### 2.8. Therapies and Bone Health

Some CDG types including PGM1-CDG and TMEM165-CDG, characterized by an undergalactosylation of the *N*-glycans have shown good responses to galactose supplementation [68,69]. Additionally, deficient import of UDP-Gal into the ER or the Golgi, required by β1,4-galactosyltransferase to elongate the growing glycan chain, has been documented in SLC35A2-CDG [70]. Although in this condition, galactose supplementation has improved glycosylation, it has not been clear whether it has had an impact on clinical manifestations of the disease including bones anomalies [70]. Apart from galactose, mannose has been utilized as a therapy in MPI-CDG [9,10] and has been shown to restore endocrine function and coagulation and alleviates enteropathy, but does not always reverse progressive hepatic involvement [71]. The long-term effect of mannose on skeletal outcomes needs careful attention. As a result of the UDP-fucose transporter defect and impaired fucosylation in the Golgi in SLC35C1-CDG, patients affected with this CDG type have been benefited from fucose supplementation [72,73]. A decreased infection rates and improved expression of E- and P-selectin ligands and restored neutrophil numbers have been documented [72,73].

Although biochemical and haematological parameters improved with these therapeutic approaches, the impact on bone tissue remains unclear. Long-term prospective observational studies will elucidate whether the bone health in CDGs improves with the nutritional supplementation and/or new therapeutic approaches including pharmacological chaperone, antisense or gene therapies (discussed in detail in [9,10]).

## 3. Conclusions

CDGs should be considered as a differential diagnosis of skeletal dysplasia, especially in multiorgan and systemic involvement. It is particularly important for CDGs with prenatal-onset skeletal dysplasia which is associated with severe clinical outcomes. Thus, early diagnosis is key to family genetic counselling.

Skeletal involvement in CDGs is underestimated and thus should be more carefully investigated and managed shortly after a diagnosis is made. In adult CDG patients screening for additional risk factors for osteoporosis should be considered. There is a scope for utilizing bone markers in monitoring of the bone tissue dynamics. The anticipated expansion in the therapeutic strategies is hoped to impact the bone health in CDGs and prevent skeletal complications.

## Figures and Tables

**Table 1:** Skeletal phenotype in various CDG types.

CDG Type	Osteoporosis	Osteopenia	Skeletal Dysplasia	Prenatal-onset Severe Skeletal Dysplasia	Limb Shortening	Growth Failure/Short Stature	Pseudodiastrophic Dysplasia	Gillessen-Kaesbach and Nishimura Skeletal Dysplasia	Desbuquois Dysplasia	Kniest Dysplasia	Schneckenbecken Dysplasia	Chondrodysplasia Punctata	Scoliosis/Kyphosis Kyphoscoliosios/	Hip Discolaction	Abnormal Digit Morphology/Brachytelephalangy	Joint Laxity	Ehlers-Danlos-like Phenotype	Abnormal Cartilage Development	Advanced Bone Age	References
PMM2-CDG	+	+	+		+	+				+										[11,12,13,14,15,16,17,18,19,20,21]
ALG12-CDG			+		+	+	+						+							[22,23,24,25]
ALG3-CDG			+	+	+	+						+								[26]
ALG9-CDG			+	+	+	+		+					+	+						[27,28,29,30]
ALG6-CDG															+					[31,32]
PGM3-CDG			+	+	+	+			+											[33,34,35,36]
COG7-CDG			+		+	+														[37,38]
COG1-CDG			+		+	+														[39]
COG8-CDG			+		+	+														[40]
CSGALNACT1-CDG			+		+	+			+							+		+		[41,42]
SLC35D1-CDG			+	+	+	+					+		+						+	[43,44]
B4GALT7-CDG		+	+			+										+	+			[45,46,47,48]
B3GAT3-CDG		+	+			+							+			+	+			[49]
TMEM165-CDG	+	+	+			+			+				+			+		+		[50]
GPI-biosynthesis defects			+												+					[51,52,53,54,55,56,57,58,59]

**Table 2:** Review of published cases of Congenital Disorders of Glycosylation who developed skeletal and bone mineral density abnormalities and their genotypes (abbreviations: n.a.—not analysed; Pt—patient; pc—percentile).

CDG Type and Related Gene (RefSeq)	Molecular Variant and Protein Change	Bone/Skeletal Manifestation	Clinical and Radiological Findings	Outcome	References
ALG12-CDG (# 607143); *ALG12* (NM_024105.4)	c.301G > A (p.Gly101Arg)/ c.437G > A (p.Arg146Gln) compound heterozygote; 2 pts (siblings)	severe skeletal dysplasia	Talipes equinovarus, short flared ribs, ulnar deviation of the wrists, delayed ossification of cervical vertebrae, generalized epiphyseal dysplasia.	very severe clinical phenotype; Pt 1 died at age 23 months due to sepsis secondary to hypogammaglobulinemia; Pt 2 died at age 67 days secondary to cardiomyopathy.	[21]
	c.117del (p.Gln40Argfs*34) / c.1001del (p.Asn334Thrfs*15) compound heterozygote; 1 pt	severe skeletal dysplasia	Interphalangeal dislocations, scoliosis, talipes equinovarus, rhizomelic limb shortening, short metacarpals, somewhat horizontal acetabular roof (similar to pseudodiastrophic dysplasia), ulnar deviation of the wrists.	pregnancy complicated by polyhydramnios; Pt died in the neonatal period.	[22]
ALG3-CDG (# 601110); ALG3 (NM_ 005787.6)	c.286G > A (p.Gly96Arg) homozygote; 2 pts	severe skeletal dysplasia	Pt 1—IUGR with short limbs. After birth—limb rhizomelic shortening with short and stubby long bones, wide metaphyses, hypoplastic cervical vertebrae, a narrow thorax with short ribs and rounded iliac wings. Chondrodysplasia punctata (CDP) in a symmetrical pattern observed in tarsal and carpal bones, patellae, hips and upper airways. Pt 2—skeletal dysplasia including short and stubby long bones with wide metaphyses, wide phalanges and metacarpals with increased metaphyseal density, narrow thorax, hypoplastic cervical vertebral bodies and rounded iliac wings.	Pt1- termination of pregnancy at 25 weeks of gestation; Pt 2 died at 12 days due to heart failure.	[26]
ALG9-CDG (# 608776); *ALG9* (NM_024740.2)	c.1173 + 2T > A, (p.?) homozygote; 3 pts (siblings)	severe skeletal dysplasia [the authors proposed the name Gilessen-Kaesbach-Nishimura dysplasia for this severe form of congenital disorder glycosylation]	P1—severe oligohydramnios and foetal biometry dated the pregnancy to 16 weeks. Pt 2—19 weeks of gestation—brachymelia, enlarged highly echogenic kidneys with moderate hydronephrosis. Pt 3—18 weeks of gestation—similar findings as in Pt 2.	Pt 1—At gestational age 26 + 3 weeks, the mother went into spontaneous labour, and the child was stillborn. Pt 2—Because of the severe kidney disease, the family decided to terminate the pregnancy at 20 + 1 weeks. Pt 3—The pregnancy was terminated at 20 weeks.	[29]
			All—decreased ossification of the frontoparietal bones, thickening of the occipital bones, deficient ossification of cervical vertebral bodies and pubic bones, round pelvis and short tubular bones with metaphyseal flaring.		
	c.1588G > A (p.Glu530Lys) homozygote; 4 pts	mild skeletal dysplasia	Pt1—hip dislocation diagnosed at birth; 6 years—all growth parameters were at the 5th pc. Delayed bone age, mesomelic brachymelia with thickening of frontal and occipital bone, mild kyphosis of thoracolumbar spine, bilateral hip dislocation, round pelvis, brachycephaly, and shortening of greater sciatic notch. Pt 2–3: mild skeletal dysplasia. Pt 4—25-day-old boy—mild skeletal dysplasia.	Mild phenotype	[30]
ALG6-CDG (# 603147); *ALG6* (NM_013339.4)	c.257+5G > A, (p.?)/ c.998C > T (p.Ala333Val) compound heterozygote; 1 pt	distal phalangeal hypoplasia	10 weeks—large, open fontanel, brachytelephalangy of the fingers 2–5, shortening of the terminal phalanx of the thumb, brachydactyly of the feet with missing ossification of most of the middle and terminal phalanges, mild metaphyseal flaring of the long bones of the lower extremities and uneven ossification of the humerus.	Mild phenotype.	[31]
	c.897_899delAAT, (p.?)/ c.494 + 2T > G, (p.?) compound heterozygote; 1 pt	distal phalangeal hypoplasia	At birth—incomplete digits on hands and feet. Bilateral reduction deformities of the distal phalanges of digits II to IV in both hands, absence of the mid and distal phalanges on digits II to IV in the feet. Distal phalangeal hypoplasia with small tapering nails, pointed distal digits, short distal phalanges and absent middle phalanx on the left second digit.	Mild phenotype.	[32]
PGM3-CDG (# 615816PGM3-CDG (# 615816); *PGM3* (NM_001199917.2)	c.821A > G, (p.Asn274Ser) homozygote; 1 pt (Pt 1) c.821dup (p.Asn274Lysfs*7)/c.1436A > G (p.Gln479Arg) compound heterozygote; 1 pt (Pt 2)	severe skeletal dysplasia with radiographic pattern of Desbuquois dysplasia	Pt 1—at birth: rhizomelic shortening of tubular bones with brachydactyly, short metacarpal and metatarsal bones and phalanges, and pectus carinatum. Bilateral exaggerated trochanter minor, coronal clefts of the caudal lumbar vertebrae, and cranial Wormian bones.	Pt1—At 4 months of age received a hematopoietic stem cell transplant (HSCT). She was globally developmental delayed (4-month stage at 1 year of age).	[33]
			Pt 2—fetal ultrasound—short limbs, small thoracic diameter. After birth—short-limbed dwarfism, brachydactyly, pectus carinatum. Short tubular bones, several phalangeal and tarsal dislocations, short femoral necks with metaphyseal beaking, and exaggerated lesser trochanters.	Pt 2—the most severe phenotype—complex neurological deterioration and died at 7 months of age from overwhelming infection.	
	c.1219T > C (p.Phe407Leu) homozygote; 2 pts *The available evidence is currently insufficient to determine the role of this variant in disease. Therefore, it has been classified as a Variant of Uncertain Significance [ClinVar Version: 15-Jun-2021].*	severe skeletal dysplasia with radiographic pattern of Desbuquois dysplasia	Pt 1— shortened limbs with hand and feet lengths <3rd pc. Spindle-shaped fingers, clinodactyly, and shortened thumbs and index. A medial deviation of the fifth toes. Wormian bones and small sella turcica, shortened long bones with large and irregular methaphyses. Pt 2—short limbs noted at 30 weeks of gestation. Several supernumerary ossification centres were noted: one next to the distal metaphysis of the humeri and another one next to the calcaneus. Autopsy showed absence of the well-defined cell columns of the proliferating and hypertrophic cartilage. The hypertrophic cell zone was reduced; the resorption zone was irregular. Primitive trabeculae were thick and misaligned.	Pt 1—died 7 days after birth of respiratory insufficiency and pulmonary hypertension.	[36]
*CSGALNACT1-CDG* (# 618870) *CSGALNACT1* (NM_001130518.2)	c.1151C > G (p.Pro384Arg)/ null (55.3-kbp deletion of exon 5–8) compound heterozygote; 1 pt	skeletal dysplasia	Shortness of long bones and a flat facial profile noted in the fetus on a routine ultrasound scan by 28 weeks of gestation; Radiographs performed at birth and at 5 months of age showed flat acetabular roofs and enlargement of the lesser trochanter with metaphyseal beaking resulting in Swedish key appearance of the femur; coronal and sagittal clefting of the vertebrae and mild advanced carpotarsal bone age constituting the mandatory pattern of Desbuquois dysplasia. 3½ years—mild micromelic and non-proportionate stature, hyperlordosis, pes planus, mild joint laxity	n.a.	[41]
	c.791A>G (p.Asn264Ser); homozygote; 1 pt (Pt 2)	skeletal dysplasia with advanced carpal bone age in infancy	Pt 1—Neonatal period—advanced carpotarsal bone age, short and plump long bones, narrow chest, coronal clefting of vertebrae, trident-shaped acetabula, monkey wrench appearance of the proximal femur. Aggravation of the phenotype with age 5 years 7 months—marked disproportionate stature, short stature, macrocephaly, brachydactyly, hyperlordosis, mild scoliosis, progressive pectus excavatum, pes planus, 2/3-toe syndactyly. Pt 2—10 years—height at 3rd pc, scoliosis, overlapping fingers, 2/3-toe syndactyly, camptodactyly of the fifth distal interphalangeal joints, clinodactyly of second finger, and limited extension of elbows and knees	n.a.	[42]
B3GAT3-CDG (# 245600); *B3GAT3* (NM_012200.4)	c.481C > T (p.Arg161Trp)/c.889C > T (p.Arg297Trp) compound heterozygote; 1 pt c.830G > A (p.Arg277Gln) homozygote; 6 pts c.419C > T (p.Pro140Leu) homozygote; 8 pts c.1A > G (p.?)/c.671T > A (p.Leu224Gln) compound heterozygote; 1 pt c.416C > T (p.Thr139Met) homozygote; 1 pt c.245C > T (p.Pro82Leu) homozygote; 1 pt c.667G > A (p.Gly223Ser) homozygote 8 pts	variable skeletal anomalies	Among 26 described cases—wide phenotypic range, including: short stature (18/23), multiple fractures 8/18, kyphoscoliosis (8/24), peculiar fingers (25/25), foot deformity (22/25), pectus excavatum/carinatum (3/19), bowing of limbs (5/19), low bone density/osteopenia (10/11), platyspondyly (1/20), radioulnar synostosis (11/13), metaphyseal flaring (3/5), iliac abnormalities (2/4), radial head subluxation or dislocation (3/5).	n.a.	[49]
SLC35D1-CDG (# 269250); *SLC35D1* (NM_015139.3)	c.125delA, (p.Ser42fs*9) homozygote; 1 pt (Pt 1) c.932G > A, (p.Trp311*)/ c.636 + 1G > T, (p.?) compound heterozygote; 1 pt (Pt 2)	Schneckenbecken dysplasia	Pt 1: 20 weeks of gestation—severe hydrops, extremely short extremities, small thorax. Radiographs showed typical findings for Schneckenbecken dysplasia, including small ilia with snail-like appearance, platyspondylia with oval vertebral bodies and general shortness of long bones with dumbbell-like appearance. The chondroosseous morphology was also typical for Schneckenbecken dysplasia. Hypercellular cartilage with scare extra-cellular matrices and absence of columnar alignment of proliferating chondrocytes.	Pt 1—Termination of pregnancy at 22 weeks.	[43]
			Pt 2: At birth—hydrops, narrow chest, the limbs were very short in all segments with bilateral clubfeet. Radiographs showed features typical of Schneckenbecken dysplasia. The ilia were short with a snail-like appearance and a flattened acetabular roof. The long bones were short and the metaphyses were abnormally wide. The spine was abnormal, with inter-pedicular narrowing, particularly in the lumbar region.	Pt 2 died of respiratory insufficiency in the immediate neonatal period.	
B4GALT7-CDG (# 130070); *B4GALT7* (NM_007255.3)	c.808C > T, (p.Arg270Cys) homozygote; 2 pts	skeletal and joint abnormalities	Pt 1: 6 months—growth retardation; 1 year—remarkable joint laxity; 2 years—head circumference at 3rd pc, weight and height significantly below the 3rd pc. Her fingers were long with prominent finger pads and lax interphalangeal joints. She had an interrupted single transverse palmer crease, lax wrists, and limited elbow movements. Her toes were long with prominent pads and partial syndactyly of 3rd and 4th toes with overriding toes. Skeletal survey demonstrated bilateral proximal radioulnar synostosis and bowing of the shafts of the radius and ulna. The clavicles were short with broad medial ends. There was anterior splaying of ribs, abnormal appearance of distal metaphyses of the humeri and ulnae, cone shaped appearance of distal phalanx of first toe, and diffuse osteopenia. Pt 2: 33 years—head circumference at 3rd pc, weight at 3rd pc, height <3rd pc. Lax joints, loose skin, and limited movement of elbow joints were observed. His fingers were long and he had a single palmar crease in the left hand. He had bilateral equinovarus deformities and there were many atrophic scars on the sides of his feet and back of his legs and hips Skeletal X-rays demonstrated findings similar to those of Pt1 in particular to the proximal radioulnar synostosis bilaterally.	n.a.	[45,46,47,48]
TMEM165-CDG (# 614727); *TMEM165* (NM_018475.5)	c.792 + 182G > A (p.?) homozygote; 3 pts (Pt 1–3)	skeletal dyplasia	Pt1—Birth weight 3250 g (50th pc), length 50 cm (25th pc), head circumference 36 cm (50th pc). 11 years—short stature (but normal head growth rate), generalized epi-metaphyseal dysplasia and joint destruction diagnosed as Desbuquois syndrome; pectus carinatum, dorsolumbar kyphosis and severe sinistroconvex scoliosis, short distal phalanges, genua vara, pedes planovalgi, joint hyperlaxity; osteoporosis and important epi- and metaphyseal dysplasia with broad metaphyses, irregular epiphyses, and thin bone cortex. The vertebrae were flattened and beaked and the phalanges were broad. Pt2—5 months—absent second-toe nails, joint hyperlaxity, right hip dysplasia.	n.a.	[6,50]
	c.377G > A (p.Arg126His) homozygote; 1 pt (Pt 4) c.376C > T (p.Arg126Cys)/c.910G > A (p.Gly304Arg) compound heterozygote; 1 pt (Pt 5)	skeletal dyplasia	Generalized osteoporosis, discrete irregular metaphyses of the long bones, discrete plathyspondyly, broad iliac wings, horizontal acetabular roofs, and subluxation of the right femur. Pt 3—birth weight of 3360 g (25th pc) and length of 49 cm (between 3rd and 10th pc). 6 years—body length—7 SDS but head circumference normal (49.9 cm, 25th pc). Radiology of the skeleton at age 1 year revealed generalized osteopenia, a J-like sella turcica, hypoplasia of the skull base, mild anterior beaking of vertebrae D11, D12, L1, and L2, broad radial and ulnar metaphyses, strongly underdeveloped carpal bones, plump and broad phalanges, horizontal acetabula, very discrete opacification of the right proximal femur epiphysis, no ossification of the left proximal femur epiphysis, broad proximal femur metaphyses, broad metaphyses and strongly underdeveloped distal femoral and proximal tibial epiphyses. Pt 4—9 years—normal growth, mild rhizomelia, no clear skeletal anomalies. Pt 5—short stature, dysplastic toenails, osteoporosis, anterior beaking of lumbal vertebrae, dysplastic vertebrae and ribs, dysplastic fourth metacarpals and metatarsals, hypoplasia of femoral heads, and kyphoscoliosis.	n.a.	[6,50]
COG7-CDG (# 608779); *COG7* (NM_153603.4)	c.169 + 4A > C (p.?) homozygote; 3 pts	variable skeletal anomalies	Pt 1—At birth—Weight 2645 g (15th pc), length 44 cm (<3rd pc), head circumference 31.5 cm (<3rd pc). Adducted thumbs, overlapping, long fingers, a simian crease, contractures of the PIP and DIP joints with ulnar deviation of the hands were observed. Pt 2—At birth—Weight 2890 g (25th pc), length 42 cm (<3rd pc), head circumference 33.5 cm (10thpc). Adducted thumbs, a simian crease, overlapping fingers with contractures of the PIP joints and ulnar deviation of the hands were observed. Pt 3—at birth—Weight 2300 g (<3rd pc), length 41 cm (<3rd pc), head circumference 31.9 cm (<3rd pc). Adducted thumbs and overlapping, long toes and fingers were observed.	Pt 1 died at the age of 9 months. Pt 2 died at the age of 7 months. Pt 3 died at the age of 8 months.	[37]
	c.169 + 4A > C (p.?)/ homozygote; 2 pts	variable skeletal anomalies	X-ray of Pt 1 lacked humerus and tibia epiphysis; Pt 2 had shoer extremities.	Died at 5 and 10 weeks, respectively	[38]
COG1-CDG (# 611209) *COG1* (NM_018714.3)	c.2665dup (p.Arg889Profs*12) homozgote; 1 pt	variable skeletal anomalies	2 months and 3 weeks—small hands and feet; 7 months—growth retardation with a rhizomelic short stature; 15 months—growth below the 5th percentile, head circumference followed the 5th percentile, progressive microcephaly; Rib fusions, rib abnormalities, vertebral abnormalities were observed.	Last follow-up at 21 months of age	[39]
COG8-CDG (# 611182); *COG8* (NM_022341.2)	c.1611C > G (p.Tyr537*) homozygote; 1 pt	variable skeletal anomalies	3 years: Small hands and feet, Hypoplasia of the first phalanx of some fingers and toes, Wide space between the first and second toes, Clinodactily of the third and fourth toes, acquired microcephay from 4 years of age.	From the age of 7, her cerebellar ataxia has worsened.	[40]
PMM2-CDG (# 212065); *PMM2* (NM_000303.3)	c.691G > A (p.Val231Met) / c.442G > A (p.Asp148Asn) compound heterozygote; 1 pt	radiographic skeletal appearances were consistent with a primary skeletal dysplasia closest to Kniest dysplasia (OMIM 156550) or spondyloepiphyseal dysplasia congenita (OMIM 183900) (type II collagen disorders).	Obstetric ultrasound examination revealed macrocephaly with shortened long bones (femur length <5th pc), lumbar lordosis; Neonatal period—short long bones with, dumbbell’’ metaphyseal expansions, generalized epiphyseal ossification delay, ovoid and anteriorly beaked vertebral bodies, hypoplastic cervical vertebrae, hypoplastic pubic bones, and bullet-shaped short tubular bones; Cervical spine MRI: elevation of the posterior arch of C1 with the occipital bone and significant spinal canal stenosis at the craniocervical junction due to a bone spur	n.a.	[7]
	n.a.	osteopenia	3 pts aged 14–27 years: Low bone density. Common clinical findings are skeletal changes including peculiar thoracic deformity and joint restriction, while a major radiological feature is diffuse osteopenia.	n.a.	[17]
	n.a.	variable skeletal anomalies	2 months—pectus excavatum, restricted extension of knees and elbows, and long fingers with camptodactyly. Diffuse osteopenia, disturbed modeling of bones: anterior widening of the ribs, thick clavicles, dorsolumbar kyphosis with slight hook-like dysplasia of the antero-inferior border of the first lumbar vertebrae. squared and flared iliac wings, horizontal acetabular roofs, and poorly formed and thickened ischial and pubic bones.	n.a.	[18]
	n.a.	variable skeletal anomalies	Osteoclerosis of the femora and tibiae with narrow medullary cavities and transverse radiolucent metaphyseal lines	n.a.	[19]
	n.a.	variable skeletal anomalies	Bone-in-bone appearance of the skeleton, with flaring of the iliac wings and sloping acetabular roof.	n.a.	[20]
PIGV-CDG *PIGV* (NM_017837.4)	c.467G > A (p.Cys156Tyr)/c.1022C > A (p.Ala341Glu) compound heterozygote; 1 pt (Pt 1) c.1022C > A (p.Ala341Glu) homozygote; 1 pt (Pt 2)	variable skeletal anomalies including brachtelephalangy	Pt 1: birth length of 53 cm (mean), weight of 4510 g (+2.0 SD), head circumference of 38.5 cm (+2.2 SD); 19 months—length 79 cm (−1.5 SD), weight 10 kg (−1.3 SD), OFC 47 cm (−1.2 SD). His fingers were short with nail hypoplasia of the first, second, and fifth digits as well as clinodactyly V. All toes showed marked nail hypoplasia. Metacarpophalangeal pattern profile provided evidence of brachytelephalangy. AP activity was elevated (907 U/L, normal range 160–360 U/L). Pt 2: Birth Weight 3,480 g (−0.7 SD), length 56 cm (+0.5 SD), and head circumference 37 cm (+1 SD). 2 years: height of 90 cm (+0.5 SD), a weight of 14.3 kg (+1.2 SD), and a head circumference of 52 cm (+1.6 SD). Hands and feet showed mild brachydactyly with mild hypoplasia of the terminal phalanges with small nails.	n.a.	[52]
PIGL-CDG *PIGL* (NM_004278.4)	c.60G > A (p.Trp20*)/c.262C > T (p.Arg88Cys) compound heterozygote 1 pt	variable skeletal anomalies including brachtelephalangy	Birth weight 2665 g (5th pc), OFC 32 cm (5th pc); 4 years: growth parameters were normal with weight, height and head circumference on the 75th pc. Mild pectus excavatum and clinodactyly involving the fifth digits, 4th and 5th toes bilaterally. X-ray hands showed deformity of the middle phalanx of the 5th finger bilaterally with the lateral aspect of the phalanx being shorter than the medial aspect.	n.a.	[53]
PIGO-CDG *PIGO* (NM_032634.4)	c.2869C > T(p.Leu957Phe) / c.2361dup (p.Thr788Hisfs*5) compound heterozygote; 2 pts (Pt 1,2) c.2869C > T (p.Leu957Phe) / c.3069 + 5G > A (p.?) compound heterozygote; 1 pt (Pt 3)	variable skeletal anomalies including brachytelephalangy	Pt1 and 2: Nail hypoplasia of the second and fourth digits and absent nail of the fifth digit. Broad hallux, small nails of the second and third toes, and aplasia of the nails of the fourth and fifth digits. Pt3: 18 months—nail hypoplasia of the second and fifth digits and clinodactyly V. Nail hypoplasia of all toes. Brachytelephalangy II to V, mostly affecting fingers II and V, and a broad distal phalanx of the thumb.	n.a.	[58]
	c.2612A > C (p.His871Pro)/ c.1810dupC (p.Arg604Profs*40) compound heterozygote 1 pt		Birth weight 2870 g, length unknown, head circumference 36.5 cm. Hypoplastic toenails at birth. 7 years: height 123 cm (SDS 0.0), weight 25.5 kg (SDS 0.6), head circumference 56 cm (SDS 2.5), mild camptodactyly of the fingers, and small hands and feet with short fingers and toes. Radiological examination of the skeleton showed a thin cortex, triangular distal toe phalanges, scoliosis.	n.a.	[59]

## Data Availability

All data generated or analysed during this study are included in this published article.

## Abbreviations

ALG3	alpha-1,3-mannosyltransferase;
ALG6	alpha-1,3-glucosyltransferase;
ALG9	alpha-1,2-mannosyltransferase;
ALG12	alpha-1,6-mannosyltransferase;
CDGs	congenital disorders of glycosylation;
COG	component of oligomeric Golgi complex;
CSGalNAcT1	chondroitin sulphate N-acetylgalactosaminyltransferase-1;
EDS	Ehlers-Danlos syndrome;
GPI	glycosylphosphatidylinositol;
GPI-BD	glycosylphosphatidylinositol biosynthesis defects;
LLO	lipid-linked oligosaccharide;
PGM3	phosphoglucomutase 3;
PMM2	phosphomannomutase 2;
SLC35D1	UDP-glucuronic acid/UDP-N-acetylgalactosamine transporter;
TMEM165	transmembrane protein 165.
